# Acute Type A Aortic Dissection Confounded by Aberrant Symptoms

**DOI:** 10.7759/cureus.18728

**Published:** 2021-10-12

**Authors:** Anderson C Anuforo, Soumya Adhikari, Eloho H Olojakpoke, Dana Aiello

**Affiliations:** 1 Internal Medicine, State University of New York (SUNY) Upstate Medical University Hospital, Syracuse, USA; 2 Medicine, State University of New York (SUNY) Upstate Medical University Hospital, Syracuse, USA; 3 Cardiology, State University of New York (SUNY) Upstate Medical University Hospital, Syracuse, USA

**Keywords:** debakey, stanford, transesophageal echocardiogram, transient ischemic attack, bentall procedure, type a acute aortic dissection

## Abstract

Acute aortic dissection (AAD) is a cardiovascular emergency that requires emergent surgical, endovascular, or medical intervention depending on the portion of the aorta implicated, as dictated by the Stanford classification, and the extent of aortic involvement. Acute chest pain radiating to the back is typically seen in AAD and may be associated with radial pulse deficits. A high index of suspicion is required to diagnose and initiate management of this emergency as early as possible. This is a report of an atypical presentation of an extensive aortic dissection identified in a young man without most of the typical risk factors, but which was promptly diagnosed and treated.

## Introduction

Acute aortic dissection (AAD) is an emergency with an incidence of about 2.53 per 100,000 person-years [1]. When left untreated, it is associated with high mortality rates of 33%, 50%, and 75% at 24 hours, 48 hours, and 14 days, respectively [2]. The most common causes of death are aortic rupture, stroke, visceral ischemia, cardiac tamponade, and circulatory failure. Furthermore, for every hour that a Stanford type A AAD is left untreated, the mortality rate increases by 1%-2% per hour [3-5]. The most common risk factor for AAD is hypertension (76.6%), while atherosclerotic disease (27%), prior cardiac surgery (16%), and inherited tissue connective diseases (ITCD) like Marfan syndrome (5%) are other common risk factors [6,7]. Patients below 40 years of age make up a minority of patients with AAD (7%) and often possess unique risk factors like Marfan syndrome or a bicuspid aortic valve based on a study employing the International Registry of Aortic Dissection (IRAD) [8]. AAD has a bimodal peak incidence with one peak before 40 years of age, usually related to connective tissue diseases, previous aortic surgery or bicuspid aortic valve, and a second peak over 60 years often associated with atherosclerosis and hypertension [9]. The management of an AAD is dependent on the magnitude of aortic and overall cardiovascular compromise and is guided by the anatomic location of the injury based on the Stanford and DeBakey classification. The Stanford classification is more frequently used to guide therapy. While a Stanford A type involves the ascending aorta and demands emergent surgical intervention, a Stanford B type involves the descending aorta and may be managed medically. Type A often presents with acute chest pain, while acute back pain is more commonly seen in Type B dissections [10]. The patient in this report presented with a history of abdominal pain, transient vision loss, atypical chest pain, dyspnea, and hemoptysis.

## Case presentation

After informed consent, we present the case of a 39-year-old Caucasian male with a past medical history most significant for schizophrenia and multiple reported fractures, who presented to the ED with a two-day history of breathlessness, orthopnea, and cough productive of pinkish frothy sputum and occasional frank blood on vigorous coughing. There was no preceding chest trauma, fever, upper respiratory symptoms, or recreational drug use. The dyspnea was mainly exertional and associated with palpitation and pleuritic chest discomfort.

Symptoms were preceded by an episode of acute abdominal pain that began a day before the presentation. The pain radiated to the flanks and upper back and was associated with nausea, transient vision loss in the left eye, and numbness in the hands. At an outside hospital one day prior, he had a CT Abdomen that reported a high stool burden and fatty liver infiltration. He received antiemetics, NSAID analgesics, and IV fluids with improvement in his symptoms. He was discharged home but presented again to the ED following the development of breathlessness and hemoptysis.

In the ED, the patient was not in acute distress and initial vital signs revealed a blood pressure (BP) of 99/42mmHg in the right arm and 98/70mmHg in the left arm. He transiently desaturated with SPO_2_ in the 80s, requiring 3L of oxygen after which the saturation improved to 97%. Subsequent vitals check revealed a BP of 112/46, PR 86/min, RR 23/min, T 37°C, and SPO_2_ of 100% on room air. Labs were significant for an elevated troponin T level of 0.11, troponin I of 0.36, and pro-BNP of 1130. Complete blood count (CBC) and basic metabolic panel (BMP) revealed a WBC count of 17,300/mm^3^ with a neutrophilic predominance and mild azotemia with a BUN of 23. Chest x-ray (CXR) showed mediastinal enlargement with diffuse patchy lung infiltrates, concerning possible venous congestion.

Cardiology service was consulted in light of elevated troponin and pro-BNP levels. The Cardiology team performed a bedside echocardiogram and suspicion for dissection was raised. Chest CT angiography performed subsequently demonstrated a Type A dissection and the patient was emergently taken to the operating room. Intra-operative transesophageal echocardiogram (TEE) showed severe aortic insufficiency (AI) as well. The patient underwent a Bentall procedure (complex graft replacement of the aortic valve, aortic root, and ascending aorta with re-implantation of the coronaries) in the operating room.

Postoperatively, he was taken to the cardiac ICU and fluid resuscitated for hypotension. Mechanical ventilation was weaned, and he was extubated successfully after 48 hours. He was started on Metoprolol tartrate post-operatively for heart rate (HR) and BP control, which was changed to Metoprolol succinate at discharge. Chest tubes were removed on postoperative day (POD) 4. Temporary epicardial pacing wires were removed on POD 6. He was discharged home on POD 7 on aspirin 325 mg for six months and metoprolol succinate 100 mg daily. A referral was placed for outpatient cardiac rehab. He was also scheduled for a follow-up CTA chest, abdomen, and pelvis, three months after discharge.

## Discussion

This case illustrates an aberrant presentation of a Stanford A aortic dissection. Stanford A is the more frequent type and is associated with a mortality rate of up to 50% without intervention [9]. Most cases of AADs are seen in hypertensive men in the sixth decade of life without genetic predispositions. The presence of risk factors raises the index of clinical suspicion of an aortic dissection. According to US studies, arterial hypertension was a major risk factor (77.8%) for AAD [8,11]. Other notable risk factors are smoking (38.9%), atherosclerosis (26.5%), and obesity (11.1%) [8]. However, our patient did not have these typical risk factors, which would have made an aortic dissection a high probability.

Furthermore, he presented to the ED with a two-day history of breathlessness, orthopnea, and hemoptysis preceded by sharp abdominal pain, which had resolved before the presentation. Breathlessness was mainly exertional and associated with palpitation and pleuritic chest discomfort. Interestingly, the typical retrosternal chest pain radiating to the back, which is seen in 84.4% of cases [10,12,13] was absent at presentation. Atypical symptoms tend to be less common and more likely to confuse the clinician and lead to misdiagnosis. They include isolated abdominal pain (30%), pulselessness (30%), heart failure (20%), cardiac tamponade (18%), Transient ischemic attack (20%), focal neurologic deficit (12%), stroke (11%), or sudden death (4%) [10-14]. Another review of the IRAD showed abdominal pain to be associated with increased mortality and to be rare as the primary presenting symptom (4.6%) [14]. Our patient did have a possible TIA associated with abdominal pain, hemoptysis, and shortness of breath, but which was atypical enough to put AAD lower on the list of differentials below an acute pulmonary embolism and acute left ventricular failure. It is important to note that the absence of classical features such as blood pressure differentials, hypotension, or neurological deficits does not rule out AAD due to their low sensitivity [15].

**Figure 1:**
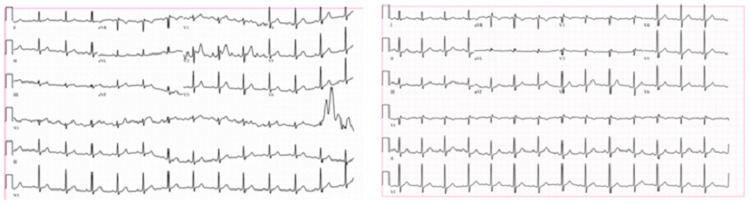
Electrocardiogram (EKG) at first ED visit (left) showing normal sinus rhythm with baseline artifact and EKG at second ED visit (right) showing sinus rhythm and T-wave inversion in V1 and V2

Troponins were also significantly elevated as expected in 50% of cases [5]. EKG (Figure 1) during the first ED visit was normal, which can be seen in 31.3% of cases [10]. However, admission EKG showed T-wave inversion in V1 and V2, in line with expected nonspecific ST-segment/T-wave changes or new Q waves usually seen [16].

**Figure 2:**
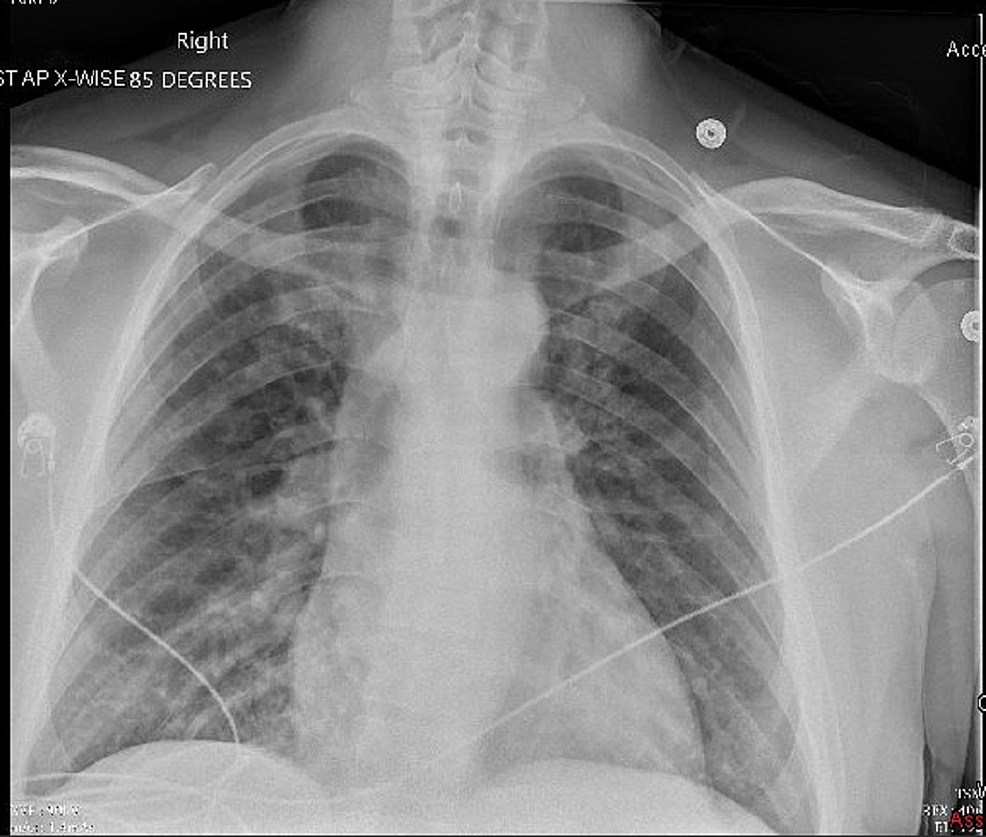
Chest x-ray (CXR) showed diffuse patchy lung infiltrates, concerning possible venous congestion or pulmonary edema with enlargement of the cardio-mediastinal silhouette and double density knob sign

Abnormal results of these two investigations would be more suggestive of an acute coronary syndrome, which could delay the diagnosis of AAD, and could lead to the initiation of incorrect antithrombotic therapy, which could lead to more complications [17-20]. Screening CXR (Figure 2) showed widening of the mediastinal silhouette and double density aortic knob, which are usually seen in about 50% of cases [20] and have a slightly higher specificity (70%) than sensitivity (67%) for AAD [21,22].

Medical therapy often involves pain control, treatment of underlying co-morbidities, and beta-blockade to reduce HR and systolic BP. Beta-blockade was initially avoided for this patient due to his normal vital signs and presence of AI as this would have prolonged diastole and worsened the AI. He was however started on beta-blockers and aspirin after the operative intervention, which is geared toward reducing shear stress on the diseased aortic segment and thrombotic phenomena, respectively. In most cases of AI secondary to Type A AAD the aortic valve is essentially normal and could be preserved via an aortic valve-sparing repair of the aortic root, however in some emergencies like our patient, aortic valve replacement is done [22]. Furthermore, aortic root replacement is indicated if dissection involves at least one sinus of Valsalva to avoid late recurrence of AI [22,23].

**Figure 3:**
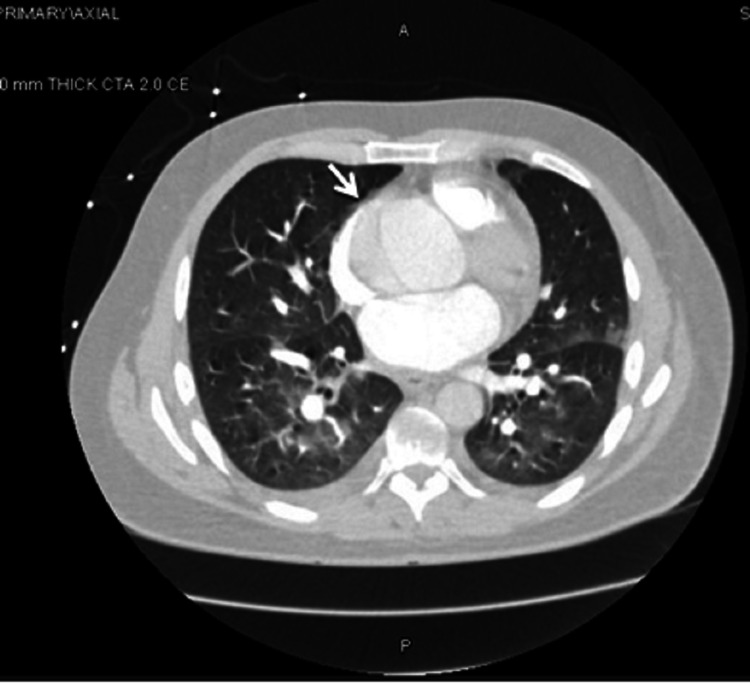
Axial CT angiography of the thorax showing aortic dissection in ascending and descending aorta, aneurysmal dilatation (arrow) of the ascending thoracic aorta, and diffuse patchy bilateral nodular infiltrates

**Figure 4:**
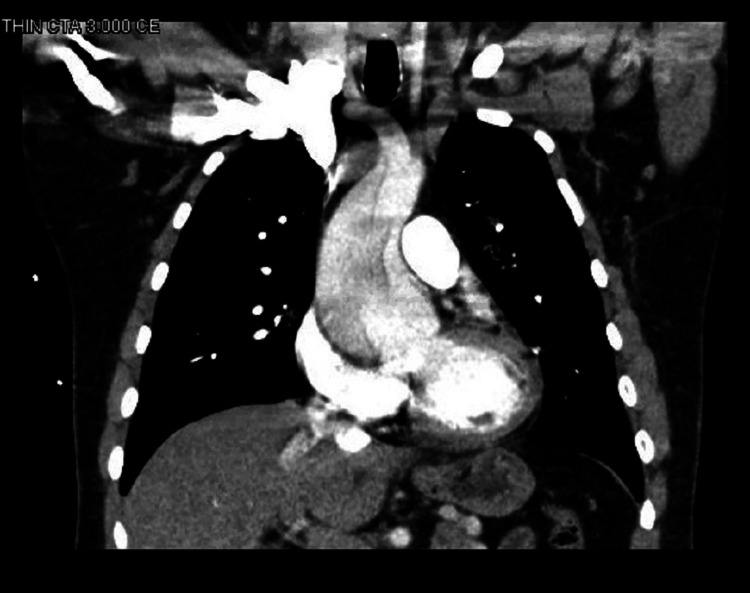
Coronal view of CT angiography of the chest showing aneurysmal dilatation of the aortic root approaching 6 cm with extensive DeBakey type 1 dissection

Imaging modalities to diagnose AAD include echocardiography and CT scans in the emergency setting and magnetic resonance imaging (MRI) for hemodynamically stable patients [18]. The bedside echo by the Cardiology department was suspicious for dissection and Figures 3, 4 show the CTA thorax performed, which ruled out a pulmonary embolism and confirmed a Type A dissection extending to the descending aorta. Intraoperative TEE was done to fully evaluate the dissection extent and showed severe AI. He had a Bentall procedure with re-implantation of right and left main coronary arteries.

Temporary epicardial pacing wires are usually placed immediately after aortic valve replacement to permit swift initiation of atrial and/or ventricular pacing in the event of a perioperative cardiac arrhythmia, which has the potential to cause significant hemodynamic instability. This is especially important in patients with right bundle branch block (RBBB) or first-degree heart blocks, at increased risk of developing a complete heart block and subsequently requiring a permanent pacemaker. This patient’s EKG changes evolved by POD 1 to include sinus tachycardia and an incomplete RBBB with ST depression and T-wave inversion in inferior and anterolateral leads. These changes were replaced by POD 2 with diffuse ST elevation in keeping with pericardial reaction to a recent surgery. The pacing wires were eventually removed on POD 6. Aortic valve surgical pathology report revealed a tricuspid valve with fibrosis and myxoid degeneration, while the ascending aorta specimen showed dissection and medial cystic necrosis.

Antithrombotic therapy after bioprosthetic valve replacement is usually individualized based on the patient’s risk profile. For patients without indications for anticoagulation, most current guidelines recommend dual-antiplatelet therapy (DAPT) for three to six months, followed by lifelong single antiplatelet therapy (SAPT) [24]; however, some studies have not found SAPT to be inferior to DAPT [25] and this patient was placed on SAPT to be reviewed after six months. Post-procedure imaging showed postoperative changes with aortic valve replacement and surgical graft extending from aortic root to mid ascending aorta. It also showed a fenestration in the dissection flap extending to the abdominal aorta with equal contrast enhancement in true and false lumens. The majority of abdominal aortic vessels arose from the true lumen with exception of the right renal artery and several right segmental lumbar arteries.

This patient's presentation with AAD at such a young age without the typical risk factors and history of multiple fractures raised concerns for an underlying connective tissue disorder; hence he was referred for outpatient genetic testing to be mediated by his Primary Care Physician. Genetic testing identified a variant of uncertain significance in COL5A1, c5026G>A, which may be associated with autosomal dominant type I or II Ehlers-Danlos syndrome (EDS) [26]. However, this particular variant has not been reported in the literature in individuals with COL5A1 related conditions but is highly conserved and predicted to disrupt protein function. Interestingly aortic dissections and aneurysms are usually associated with the vascular subtype (type IV) of EDS, which usually results from pathogenic variants in COL3A1 often with glutamic acid to lysine substitutions (Glu>Lys) [27]. Consequently, it is currently unclear if the mutation contributed to his vascular disorders or not, and further research like cohort and case-control studies are required to clarify this.

## Conclusions

Atypical presentations of AAD are rare and can frequently mislead clinicians in the diagnosis and management of the patient's presenting symptoms. Misdiagnosis based on the absence of “typical” epidemiologic features or clinical symptoms may prove fatal and a high index of suspicion is required when AAD disguises with atypical symptoms. We hope that this report further increases awareness of the less common presentations of AAD and generates further research about the less common mutations that could be involved in the vascular subtype of EDS.
